# A Comparative Study to Evaluate Difficult Intubation Using Ratio of Patient Height to Thyromental Distance, Ratio of Neck Circumference to Thyromental Distance, and Thyromental Height in Adult Patients in Tertiary Care Centre

**DOI:** 10.5152/TJAR.2022.22077

**Published:** 2023-04-01

**Authors:** Sukhdev Rao, Naveen Paliwal, Sunil Saharan, Pooja Bihani, Rishabh Jaju, U.D. Sharma, Monish Sharma

**Affiliations:** 1 Department of Anaesthesiology, Dr. S.N. Medical College, Jodhpur, 342001, India; 2 Department of Anaesthesiology, AIIMS, Deoghar, Jharkhand, India

## Abstract

**Objective:**

Patients’ airway assessment is one of the foremost responsibilities of every anaesthesiologist. Several preoperative predictive methods have been studied by various authors to find the best difficult airway predictor. We conducted this study to compare 3 methods to predict the difficulty of laryngoscopic endotracheal intubation viz ratio of patient height to thyromental distance, ratio of the neck circumference to thyromental distance, and thyromental height in adult patients.

**Methods:**

This prospective observational study was conducted on 330 adult patients, American Society of Anaesthesiologists (ASA) status I and II, aged 18-60 years of either sex, weighing 50-80 kg scheduled for elective surgeries under general anaesthesia. Patients’ height, weight, and body mass index were recorded, and thyromental distance, neck circumference, and thyromental height were measured preoperatively. Laryngoscopic view was graded according to the Cormack–Lehane grade. Predictive indices and optimal cutoff values were calculated using the receiver operating characteristic curve analysis.

**Results:**

Difficulty in laryngoscopic endotracheal intubation was encountered in 12.42% of patients. The sensitivity, specificity, positive predictive value, negative predictive value, and area under the curve for thyromental height were 100%, 95.2%, 75.54%, 100%, and 0.982, respectively; for the ratio of patient height to thyromental distance the respective values were 75.6%, 72.7%, 28.18%, 95.45%, and 0.758 and for the ratio of the neck circumference to thyromental distance, the values were 82.9%, 65.4%, 25.37%, 96.42%, and 0.779, respectively. There was no statistically significant difference to predict the difficulty of laryngoscopic intubation between any of them (*P* < .05).

**Conclusion:**

Among these 3 parameters, thyromental height was found to be the best preoperative method to predict difficult laryngoscopic endotracheal intubation with the highest predictive indices and area under the curve. The ratio of the neck circumference to the thyromental distance method was found to be a more sensitive and useful method than the ratio of patient height to the thyromental distance method to predict the difficulty of laryngoscopic endotracheal intubation.

**Keywords:**

Body mass index, endotracheal intubation, laryngoscopic, ROC curve, thyromental height

**Main Points**

- Thyromental height test (TMHT) is a simple bedside test to predict difficult airways in the preanaesthetic checkup and is measured as the distance between the anterior border of mentum and anterior border of thyroid cartilage. It can be measured without using any specific instruments.
- In comparison to the different difficult airway predictors, TMHT has the highest predictive indices which include sensitivity, specificity, positive predictive value, negative predictive value, and area under the curve.
- Thyromental height is measured in the neutral head position unlike other predictive tests; therefore, this objective measurement test can be used in patients with limited neck movements and unstable cervical spine.

## Introduction

Airway assessment constitutes a pivotal portion of the preanaesthetic checkup for identification of patients, in whom we may encounter difficult intubation/ventilation. According to a survey by All India Difficult Airway Association, the incidence of difficult intubations varies between 8% and 13% in the intensive care units, emergency department, and out-hospital situations and between 1.5% and 13% during general anaesthesia.^1,2^ Similarly, according to the National Audit Project 4, encountered difficult or failed intubations or cannot ventilate cannot intubate situations accounted for 42% of all adverse events under general anaesthesia.^3^

Preoperative airway assessment should predict and identify potential problems which we may encounter in the operating room and allow us to develop an appropriate management plan prior to the procedure. Several studies have evaluated different models to predict difficult airway, but no single model has proven to be efficacious in identifying the problems universally.^4–6^

Many readily available bedside tests requiring no specific equipment have been developed, and still comparative systemic evaluation of these is lacking especially in the Indian population. This prospective observational study was designed to compare the ratio of height to thyromental distance (RHTMD), neck circumference to thyromental distance (RNCTMD), and thyromental height (TMHT) in the evaluation of difficulty of laryngoscopic endotracheal intubation in adult patients. We hypothesized that TMHT would be a better difficult intubation predictive test compared to other tests used in our study. The primary objective of the study was to determine the sensitivity of TMHT as a difficult intubation predictor during the pre-anaesthesia evaluations. We also determined the sensitivity, specificity, positive predictive values, and negative predictive values of RHTMD and RNCTMD along with the optimal cutoff values for each of these parameters using area under the curve (AUC) of the ROC curve.

## Methods

Institutional Ethical Committee approval was taken, and the trial was registered in Clinical Trial Registry of India (CTRI/2019/12/022445). We conducted this prospective observational study enrolling 330 adult patients of either sex, aged 18-60 years, weighing 50-80 kg of ASA I and II status posted for elective surgeries requiring general anaesthesia with endotracheal intubation. Patients with any history of previous surgery; those who refused to participate, were edentulous, were pregnant, had any upper airway facial abnormality or pathology, had tumour, and had midline neck swelling; those requiring rapid sequence induction or awake intubation; those with burns, radiation, or trauma to upper airway; or those who were unable to stand erect were excluded from the study.

A detailed pre-anaesthetic checkup was done, written informed consent was obtained, demographic data [age, gender, height, and body mass index (BMI)] were collected, and airway assessment measurement was carried out. Height (cm) was measured with the patient standing barefoot against a solid wall. Measurement for the thyromental distance (TMD), in centimetres, was done keeping the mouth closed with head in full extension, from the thyroid notch to the chin. We measured the neck circumference (NC) in centimetres at the level of the cricoid cartilage. The RHTMD and RNCTMD were then derived.

The TMHT was measured using a depth calliper at horizontal level, from the anterior border of the chin (the mental protuberance) to the anterior border of thyroid cartilage, keeping the patient supine on a flat surface with head and neck in the neutral position and mouth closed.

Induction of anaesthesia was carried out in all participants as per standardized hospital anaesthesia protocol. Optimal position for intubation (sniffing position) was achieved by placing a pillow/doughnut under the occiput during intubation. Evaluation of difficult laryngoscopy, endotracheal intubation, and the grading of laryngoscopic glottic view according to the Cormack and Lehane grading system were performed by an anaesthesiologist, having an experience of ≥2 years.^7^ Macintosh laryngoscope blades, sizes 4 and 3 for males and females, respectively, were used to guide intubation. Visibility of larynx corresponding to Cormack–Lehane (CL) grades I or II were considered as easy laryngoscopic views, whereas CL grades III or IV were considered as difficult laryngoscopic views followed by grouping of both views in easy and difficult groups, respectively.

### Statistical Analysis

All statistical analyses were performed by using version 22.0 of Statistical Package for Social Sciences (SPSS) software package (SPSS Inc., Chicago, Ill, USA). In previous studies, the sensitivity of TMHT test ranged from 75% to 92%.^4,8–11^ Sample size was calculated with expected minimum sensitivity of TMHT as 75% and the expected prevalence of difficulty of laryngoscopic difficult intubation as 13%. Confidence interval was set at 95% with a relative allowable error/precision of 20%. A sample size of minimum of 247 patients was obtained by prospective power analysis, but prospectively we collected and analysed the data of 330 participants.

Yates chi-square test, Fisher’s exact test, and unpaired *t*-test were used for the comparison of data. Data were summarized as mean ± SD for continuous variables and as numbers and percentages for categorical variables. Measured and derived values of the study methods of airway assessment done preoperatively and the CL grades were used to determine the predictive indices such as sensitivity, specificity, positive predictive value, and negative predictive value for each method. The area under the receiver operating characteristic (ROC) curves was used to calculate the optimal cutoff point and discriminative comparison of the tests.

## Results

**Figure 1.**
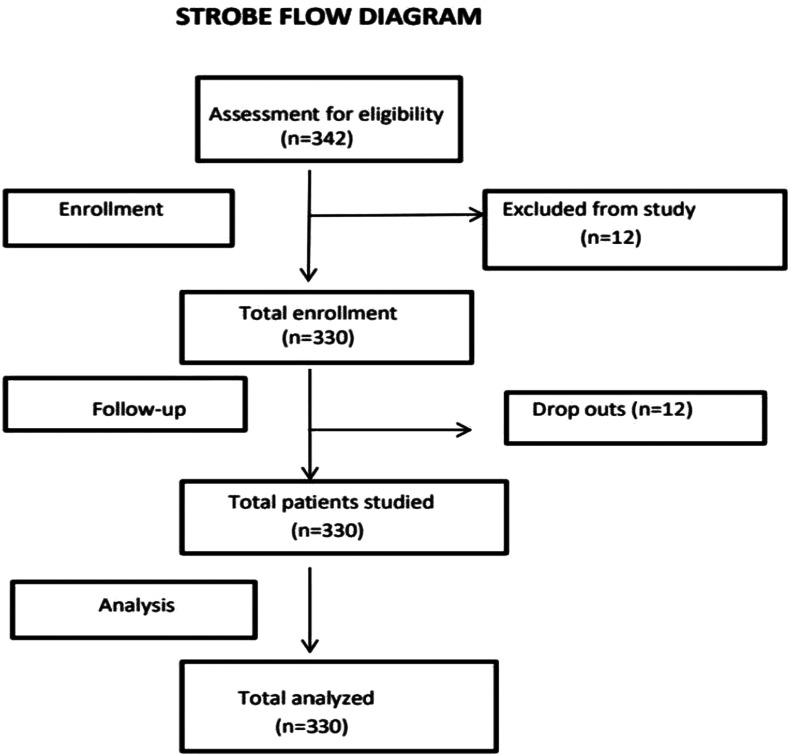
STROBE (Strengthening the Reporting of Observational Studies in Epidemiology) flow diagram.

A total of 330 enrolled patients were analysed in this study (Figure 1). The demographic profile (height, weight, BMI, and gender) of all study participants is shown in Table 1. Among the study participants, most of the participants in whom difficulty of intubation was observed belonged to age 41-50 years and had a height of 141-150 cm, weight of 71-80 kg, and BMI of ≥30. No difference was found with respect to gender between both groups.

Among these participants, 41 (12.42%) patients had CL grade either III or IV and were intubated with help of either external laryngeal manipulation or stylet or bougie. No cases of failed intubations were encountered in this study.

**Figure 2.**
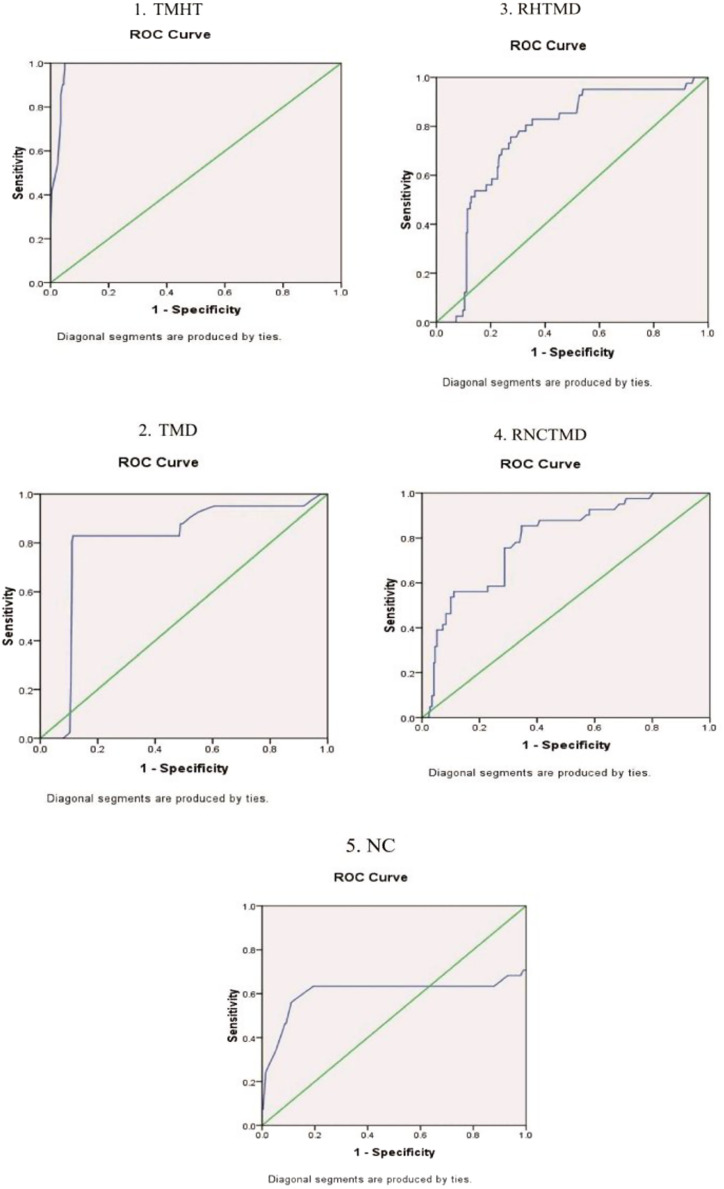
Plotting of receiver operating characteristic (ROC) curve for individual preoperative airway predictive method. NC, neck circumference; RHTMD, ratio of patient height to thyromental distance; RNCTMD, ratio of the neck circumference to thyromental distance; TMD, thyromental distance; TMHT, thyromental height.

Sensitivity, specificity, PPV, NPV, and AUC of our study variables in predicting the difficulty of endotracheal intubation are presented in Table 2, and the ROC curve showing the pairwise comparison of all the tests are shown in Figure 2.

On discriminate analysis of ROC curves, the calculated optimal cutoff values for TMHT, RHTMD, RNCTMD, TMD, and NC were 5.65 cm, 21.89, 4.9, 7.0 cm, and 39.75 cm, respectively, and the AUC was found to be maximum for TMHT (0.982) and minimum for NC (0.605). The TMHT has been shown to have the highest sensitivity of 100% with 95% CI (0.970-0.994) and the highest specificity (95.2%) and the highest PPV and NPV (75.54% and 100%, respectively) for predicting difficult intubation.

Among all these tests, NC had the lowest sensitivity (56.1%) and NPV (93.45%), while RNCTMD had the lowest specificity (65.4 %) and PPV (25.37%).

## Discussion

To facilitate difficult airway management and to decrease the likelihood of adverse outcomes, systematic preoperative identification of difficult airway is an essential component of pre-anaesthetic evaluation. In the literature, reported incidence of difficult intubation ranges from 1.5% to 13%.^2,3^ The potential adverse outcomes associated with mismanagement/failure to manage difficult airways include mortality, neuronal injury, and cardiopulmonary arrest, proceeding towards surgical airway and airway trauma.^12^ Despite the availability of multiple bedside assessment tests, the accuracy of 1 particular test to identify patients at risk of difficult intubation/facemask ventilation is limited.

Incidence of laryngoscopic guided endotracheal intubation as per CL grading in our study was found to be 12.42%. Several factors may be attributed for this large reported incidence in the literature, such as ethnic differences among populations, variable methods of laryngoscopy and endotracheal intubation, use of Sellick manoeuvre, external manipulation of larynx, type and size of blades used, number of laryngoscopy attempts, different criteria used to define difficult laryngoscopy and intubation, and varying skill of anaesthesiologists.

A difficult airway predictor test should have high sensitivity; high specificity, and high PPV and a low false-negative prediction value. A high false-negative predictive value of the test may be dangerous as patients with potential difficult airways may be missed, and we stand with an unprepared plan to manage it. A high false-positive predictive value of the test is also alarming as unnecessary manpower, time, and resources will be consumed for alternative approaches so as to manage the airway and may lead to patient discomfort. A meta-analysis by Roth et al^13^ to determine the diagnostic accuracy of commonly used bedside tests to assess adult patients at risk of difficult intubation has reported low sensitivity of some of the tests. Their study results revealed that the Mallampati test, the modified Mallampati test, and the upper lip bite test (ULBT) had a low sensitivity and that 3-5 patients out of 10 would be missed by these tests, and with such high false-negative results, anaesthesiologists might encounter trouble during induction of anaesthesia.

The TMHT which is a simple bedside airway assessment test, and it was found to be the most sensitive (100%) and the most specific (95.2%) and to have the highest PPV (75.54%) and highest NPV (100%), whereas RNCTMD was found to be the least specific (65.4%) and have the lowest PPV (25.37%) and NC was found to be the least sensitive (56.1%) and have the lowest NPV (93.45%) among all the methods studied. The optimal cutoff value of TMHT for predicting difficult laryngoscopic intubation calculated from the ROC curve was found to be ≤5.65 cm. In other studies, the optimal cutoff values for TMHT have been reported to range from 4.75 cm to 5.3 cm.^4,8–10^ The TMHT indirectly predicts the degree to which the mandible can be protruded, space in submandibular region, and the anterior position of larynx. First proposed by Etezadi et al.^9^ studies have shown TMHT to be an easy and accurate test to predict difficult airway compared to conventional tests such as MPG, Upper Lip Bite Test (ULBT), and TMD. 

The AUC denotes the diagnostic accuracy and discriminative power of a particular test, with values ranging from 0 to 1. A value between 0.6 and 0.7 is acceptable, while a value more than 0.9 is considered outstanding for any particular test.^14^ In our study, the largest value of AUC (0.982) was found with TMHT among all the study predictors, while RNCTMD had the lowest AUC (0.605). The highest AUC of the ROC curve was also reported for TMHT (0.92) in a study by Rao et al.^8^

The optimal cutoff of RHTMD to predict difficult laryngoscopic intubation was calculated to be ≥21.89 and the sensitivity of our study was found to be less than the previous study (88.4% with a cutoff value ≥18.5)^7^ due to the higher cutoff value of our study (≥21.89), which is probably due to anthropometric differences between the population groups. Higher cutoff value of our study participants decreased the number of true-positive patients and hence decreased the sensitivity in our study. The optimal cutoff of RNCTMD for difficult laryngoscopic intubation was calculated to be ≥4.9 in our study.

The optimal cutoff of TMD for predicting difficult laryngoscopic intubation was calculated to be ≤7.0 cm which was comparable with the previous study (cutoff ≤7.0).^15^ Thyromental distance was found to be a highly specific method to predict the difficulty of laryngoscope-guided endotracheal intubation. Majority of the patients with difficult intubation had TMD values between 5.1 and 7.0 cm, i.e., lesser the TMD, the higher the chances of facing difficulty during endotracheal intubation. The optimal cutoff value of NC for predicting the difficulty of laryngoscopic intubation was calculated to be ≥39.75 cm. The NC was found to be a highly specific method to predict the difficulty of laryngoscopic intubation, which was found comparable with a previous study (89.07%, with a cutoff value >39.5 cm).^13^ Among all enrolled patients, the mean NC was found to be 38.18 ± 1.82 cm, which is comparable with the mean NC measured in a previous study (37 ± 4 cm).^16,17^ Majority of the patients with difficult intubation were found to have NC as ≥41.0 cm, i.e., greater the NC, higher the chances of facing difficulty during endotracheal intubation.

Though individual bedside tests have proven to be poor predictors of difficult intubation when used alone, in clinical practice if a test has good sensitivity and specificity, it can be used to predict difficult intubation in emergency situation as a useful bedside test. In the present study, we found TMHT to be a highly predictive, discriminative, and a useful screening method among all preoperative assessment methods studied. The other major advantage of using TMHT is that it is measured with head in the neutral position unlike TMD which is measured in an extended head position.^18^ Therefore, this objective measurement test can be used in patients with limited neck movements and unstable cervical spine. Since TMHT is a highly sensitive, specific, and simple method and can be easily performed with the patient lying supine, it may be very useful in cases of emergency intubation in critically ill patients lying on the bed to predict difficult intubation correctly compared to other available clinical methods like inter incisor gap, ULBT, Mallampati grade, etc. which require patient cooperation, hence, making them unsuitable in critically ill patients requiring endotracheal intubation.

The strength of this study are as follows: a larger sample size, all preoperative measurements were performed by a single anaesthesiologist, decreasing inter-observer bias, and better assessment of laryngoscopic view as all laryngoscopies were performed by an anaesthesiologist having experience of more than 2 years. Though we also acknowledge the few limitations of our study like feasibility of these methods particularly the RHTMD and RNCTMD; in patients with limited neck movement, unable to stand erect, spinal injury, trauma, critically ill patients in emergency department and pre-hospital settings. We did not compare the utility of these tests in pregnant or paediatric population. Our study population included all adult patients weighing in the range of 50-80 kg, and we did not exclude patients on the basis of BMI, which may act as an effect modifier.

We conclude that the TMHT, a non-invasive and easy-to-perform bedside screening method to anticipate difficult intubation during routine pre-anaesthetic checkups, had higher sensitivity, specificity, PPV, and NPV scores in comparison to RHTMD and RNCTMD. It may be used as a preferred tool to predict difficult intubation in emergency situations, with patients in lying down positions along with cervical spine injuries, as it can be performed in a neutral head and neck position.

## Figures and Tables

**Table 1.** Distribution of Demographic Data

Variables	Range	Mean ± SD			*P*
		Difficult Group		Easy Group	
Age (years)	18-60	46.82 ± 9.82		34.54 ± 12.93	<.001
Weight (kg)	52-78	66.26 ± 8.06		63.96 ± 6.08	.030
Height (cm)	141-178	155.90 ± 10.32		167.86 ± 4.76	<.001
BMI (kg m2^-1^)	17.99-34.71	27.62 ± 5.04		22.68 ± 1.79	<.001
Gender, n (%)	Male: 163 (49.4%)		Female: 167 (50.6%)		.802

BMI, body mass index.

**Table 2.** Statistical Analysis of Individual Preoperative Airway Predictive Method

Test	Cutoff	Sensitivity (%)	Specificity (%)	PLR	NLR	PPV (%)	NPV (%)	AUC
TMHT (cm)	5.65	100	95.2	20.83	0.00	75.54	100	0.982
RHTMD (cm)	21.89	75.6	72.7	2.76	0.33	28.18	95.45	0.758
RNCTMD (cm)	4.9	82.9	65.4	2.39	0.26	25.37	96.42	0.779
TMD (cm)	7.0	82.9	88.6	7.27	0.19	50.75	97.33	0.801
NC (cm)	39.75	56.1	88.9	5.05	0.49	41.81	93.45	0.605

PLR, positive likelihood ratio; NLR, negative likelihood ratio; AUC, area under the curve; NC, neck circumference; NPV, negative predictive value; PPV, positive predictive value; RHTMD, ratio of patient height to thyromental distance; RNCTMD, ratio of the neck circumference to thyromental distance; TMD, thyromental distance; TMHT, thyromental height.
